# Promoting Cell Proliferation Using Water Dispersible Germanium Nanowires

**DOI:** 10.1371/journal.pone.0108006

**Published:** 2014-09-19

**Authors:** Michael Bezuidenhout, Pai Liu, Shalini Singh, Maeve Kiely, Kevin M. Ryan, Patrick A. Kiely

**Affiliations:** 1 Department of Life Sciences, and Materials and Surface Science Institute, University of Limerick, Limerick, Ireland; 2 Materials and Surface Science Institute and Department of Chemical and Environmental Sciences, University of Limerick, Limerick, Ireland; 3 Stokes Institute, University of Limerick, Limerick, Ireland; University of Massachusetts Medical, United States of America

## Abstract

Group IV Nanowires have strong potential for several biomedical applications. However, to date their use remains limited because many are synthesised using heavy metal seeds and functionalised using organic ligands to make the materials water dispersible. This can result in unpredicted toxic side effects for mammalian cells cultured on the wires. Here, we describe an approach to make seedless and ligand free Germanium nanowires water dispersible using glutamic acid, a natural occurring amino acid that alleviates the environmental and health hazards associated with traditional functionalisation materials. We analysed the treated material extensively using Transmission electron microscopy (TEM), High resolution-TEM, and scanning electron microscope (SEM). Using a series of state of the art biochemical and morphological assays, together with a series of complimentary and synergistic cellular and molecular approaches, we show that the water dispersible germanium nanowires are non-toxic and are biocompatible. We monitored the behaviour of the cells growing on the treated germanium nanowires using a real time impedance based platform (xCELLigence) which revealed that the treated germanium nanowires promote cell adhesion and cell proliferation which we believe is as a result of the presence of an etched surface giving rise to a collagen like structure and an oxide layer. Furthermore this study is the first to evaluate the associated effect of Germanium nanowires on mammalian cells. Our studies highlight the potential use of water dispersible Germanium Nanowires in biological platforms that encourage anchorage-dependent cell growth.

## Introduction

Nanowires of Group IV elements (Si, Ge) have attracted significant interest due to their size dependent physical properties. They have well established uses in Field Effect Transistors [1], as lithium ion battery anodes [2], and as components of photovoltaic cells [3]. Common group IV inorganic materials have also shown advantageous results for biomedical applications [4]–[8]. Most of this work has been done using silicon nanowires as they integrate well with complementary metal oxide semiconductor (CMOS) systems. As well as this, silicon nanowires play a central role across biomedical platforms including; single cell probing [1], gene delivery mechanisms [9], cell adhesion platforms [5], [8], enhanced biomarker detectors [10] and as carriers for other nanomaterial's, which can promote hypothermia of cancer cells [11].

Silicon nanowires have been shown to support mammalian tissue [6], [7]. Post modifications of the wires render them compatible as synthetic bone coatings [12]. The use of nanowires in biological applications requires that they be non-toxic and must not adversely affect biological activities [13]. A complication with most synthetic nanomaterials is that they contain heavy metal catalysts or functional ligands which although are required for material dispersibility, can be adversely toxic to cells. Several studies on the surface chemistry of silicon nanowires have highlighted the importance of the functional group interaction with the cellular environment [7], [9], [14]. Silicon nanowires with an oxide surface functional group have decreased adverse effects on biological reactions when compared to silicon nanowires with other common ligands with hydrophilic head carboxyl groups [14]. The orientation of the material can also directly impact the behaviour of the cellular response, for example, vertically aligned wires and suspended wires have been shown to differentially affect cell adhesion, cell spreading and overall cell morphology [6], [7], [9]. The formation of a protein corona on the surface of the nanomaterials can determine the possible biological interactions different materials may have in a cellular setting [15], [16]. Aspect ratio plays an important role in cellular repose, work done on CeO2 nanowires and rods highlight the relationship between aspect ratio and frustrated phagocytosis and lysosome rupture [17]. These works highlight that any nanomaterial for biomedical application use must be considered for its orientation and surface chemistry to assess the conditions which render it biocompatible. However the downstream effects of the material must also be evaluated for environmental impact if they are to be commercially exploited [18]–[22].

Studies into the use of germanium nanowires in biological applications have been neglected. However, it is documented that Germanium nanoparticles (GeNPs) of 4.2±1.2 nm display cytotoxicity in CHO cells [23]. The GeNPs at low concentrations (<5 µM) promote necrotic cell death. In order to make GeNPs water dispersible, ligands such as alkyamines are employed on their surface [23]. However, as seen with silver nanoparticles [24], the GeNPs may only be acting as carriers of the toxic ligand. The use of ligands brings its own complications; studies indicate that most ligands that promote water dispersibility are toxic to cells, stimulating cell damage and growth arrest. Polyethylene glycol (PEG) has been used to render germanium nanowires polar solvent dispersible, through micelle formation [25], however, little has been done to assess the cytotoxicity associated with that treatment.

Recently we developed a route to germanium nanowires using the vapour phase of a high boiling point solvent (HBS) as a growth medium facilitating the high density growth of ligand free, seedless pristine wire [26]–[28]. The HBS method produces three distinct populations of wires categorised based on their fault distribution [28]. Wires are typically between 7–40 nm in diameter and micrometres in length giving them a high aspect ratio with population distributions based on their respective faults. These pristine wires have a thin ∼5 nm oxide coating, which unlike silicon oxide is not stable in an oxidising environment [29], [30]. The unstable oxide readily breaks down to form Ge(OH)_4_ in an aqueous environment [29]. Passivation methods require specialized equipment, elevated temperatures and pressures under controlled atmospheres. This has typically been done using harsh chemicals such as HCl or HF. The passivation process can either chlorinate the surface of the wire (Ge-Cl) for later Grignard reaction, or (Ge-H) terminated surfaces can be used to promote hydrogermeylation with some alkene or alkyne [31], [32]. These approaches solve issues for the device industry but offer little solution for biocompatibility. Work on silicon oxide surfaces with amino acids and biomolecules have indicated that amino acids on the surface of the metal show strong adhesion selectivity [33], [34]. The polar charged amino acids have shown strong selectivity for silicon oxide with a pH dependency.

Here we describe a facile approach to make seedless and ligand free Germanium nanowires (synthesised in a high boiling point solvent) water dispersible. Our findings indicate that not only have we produced a water dispersible Germanium nanowire, we have also produced a non-toxic germanium nanowire that exhibits exciting properties such as a ligand free surface chemistry and the presence of an etched surface. We include a comprehensive characterisation of the properties of the nanowires together with a series of complimentary and synergistic cellular and molecular approaches to highlight the potential use of these wires in biological platforms to promote cell adhesion and proliferation.

## Materials and Methods

### Synthesis of Germanium nanowires

Germanium nanowires were synthesised by the high boiling point method as previously described [26]–[28]. In a typically reaction Squalane (7 mL, ≥99% Aldrich) is added to a long neck round bottom flask. The flask is heated under vacuum to 125°C for 1 hr to remove any residual moisture. The flask is then purged with Argon and ramped to 425°C and allowed to stabilise. Diphenylgermane (DPG) (0.2 mL, >95% Gelest) is then rapidly injected into the refluxing Squalane. Reactions are allowed to run for 15 min and then cooled. Toluene is added to flask and the wires are dispersed with the aid of sonication. Several washes are performed to insure the purity of the wires before samples are used for post treatment.

### Post treatment and characterisation of Germanium nanowires

Pristine wires suspended in toluene are centrifuged in weighed glass vials at Beckman Coulter microfuge 22R for 20 min to form a pellet. The wires are left at room temperature for 24 hrs to remove any excess toluene. Vials are then reweighed and total nanowire mass is calculated. Since the surface is ligand free, this is the true mass of the wires. Samples are then sterilized under UV for 40 min, before adding D-Glutamic acid (0.008 g/L, ≥99% Sigma) which had been sterilized by filtering through a 0.22 µm disposable filter. Germanium nanowires are then sonicated for 5 min in EMAG 20 HC sonicator to produce a (purple/brown) clouded dispersion of nanowires. The surface morphology of the nanowires was characterised using a transmission electron microscope (TEM) JEOL-2010; a scanning electron microscope (SEM) Hitachi SU-70 at 10 KV; and X-ray photoelectron spectroscopy (XPS) Kratos AXIS-165.

### Cell culture

MCF-7 cells (human epithelial breast adenocarcinoma, originally sourced commercially from ATCC) and L929 cells (murine aneuploid fibrosarcoma, originally sourced commercially from ATCC) were cultured in DMEM media supplemented with 10% Fetal bovine Serum FBS (Sigma), 1% L-glutamine (Sigma), 1% Penicillin streptomycin (Sigma) at 37°C in a humidified incubator of 5% CO2 as described previously [35]–[37], For all experiments, MCF-7 cells were seeded at 27,000 cells/well (unless otherwise indicated) in a 96 well plate and incubated with nanowires for 24 Hr before endpoint testing. L929 were seeded at 10,000 cells/well in a 96 well plate and incubated with nanowires for 24 Hr before endpoint testing was performed. Where used, glass coverslips were coated with nanowires at high concentrations mixed into 10 ng/µL collagen (collagen type 1 C7661 Sigma).

### MTT Assay

Cell metabolic activity was assessed using Millipore MTT (3-(4,5-dimethylthiazol-2-yl)-2,5-diphenyl tetrazolium kit (Millipore CT02). For all MTT assays and experiments, MCF-7 cells were seeded at 27,000 cells/well (unless otherwise indicated) in a 96 well plate and L929 were seeded at 10,000 cells/well in a 96 well plate before incubating with wires and endpoint testing. Cells were exposed to nanowires at indicated concentrations before incubation for 24 hr and endpoint analysis as per kit instruction. All readings were performed on an ELx808 absorbance microplate reader BioTek, wavelength of 570 nm and a reference wavelength of 630 nm. The outer wells of the plate were excluded from testing and filled with sterile PBS to reduce the edging effect.

### WST assay

Cell viability was recorded using the cell proliferation reagent WST-1 (Roche 11644807001), in which the tetrazolium salt is cleaved to form a soluble formazan. For all WST assays and experiments MCF-7 cells were seeded at 27,000 cells/well (unless otherwise indicated) in a 96 well plate and L929 were seeded at 10,000 cells/well in a 96 well plate. Cells were exposed to nanowires for 24 Hr and endpoint analysis was performed as per kit instructions all readings were performed on a Thermo scientific multiskan FC microplate photometer, using wavelengths between 420–480 nm with a reference of 600 nm. The outer wells of the plate were excluded from testing and filled with sterile PBS to reduce the edging effect.

### Cell proliferation assay

Cells were cultured in DMEM media supplemented with 10% Fetal bovine Serum FBS (Sigma), 1% L-glutamine (Sigma), 1% Penicillin streptomycin (Sigma) at 37°C in a humidified incubator of 5% CO2 as described previously.MCF-7 and L929 cell were seeded in a 24 well plates with 10,000 cell per well in multiple wells and grown in triplicate plates for 4 days. To monitor the cell growth of cell exposed to 2 µM, 4 µM and 7 µM of water dispersed germanium nanowires relative to an untreated control, attached cells were removed in triplicate using 100 µL of trypsin-EDTA for 5 min. After trypsinizing 200 µL of complete media was added and mixed thoroughly before 50 µL sample was then taken from each triplicate and mixed with equal volume of trypan blue exclusion assay in a 96 well plate. Trypan blue exclusion assay samples were then pipetted several times to insure even mixing of the sample before 10 µL of each sample was loaded onto the hemocytometer and cell counts were performed. Data is presented as a mean and S.D of counts in triplicate wells (n = 3).

### Western blotting

Cells were cultured on 10 cm dishes, with a typical seeding of 1.5 million/plate. Nanowires were either coated over the surface of the plate or suspended into the culture media and allowed to precipitate onto the cells. Cells were cultured for 24 hr before lysing by scraping into lysis buffer (20 mm Tris, 50 mm Nacl, 50 mM NaF, 1% Npa O, Aprotinin 0.15 U/ml, Leueptin 20 mM, PepstatinA µ1 g/ml, PMSF 2 mM, Na3Vo4 0.5 mM), incubating on ice for 10 min before being centrifuged for 15 min at 14,000 rpm on a Beckman coulter microfuge 22R centrifuge. The protein concentration of the samples was determined using the Bradford assay. Equal protein amounts were resolved on a 12% SDS polyacrylamide gels before being transferred to a nitrocellulose membrane. The membrane was then blocked for 1 Hr at room tempratue in Tris-buffered saline with 0.05% Tween 20 (TBS-T) and 5% milk (W/V). The primary antibodies were all incubated overnight at 4°C, the primary antibodies used were pERK (Cell Signaling Tec.), actin (Santa Cruz), RACK1 (Santa Cruz), FAK (Santa Cruz), Secondary antibodies were incubated at room temperature for 1Hr, the use of Alexa Flour 680 nm and 800 nm coupled anti-rabbit and anti-mouse antibodies were used for detection (Li-COR biosciences Cambridge, UK). Western blot analysis was performed on an ODYSSEY inferred imaging system (Li-COR Biosciences, Cambridge, UK).

### Fluorescent imaging

Glass cover slips (VWR) were autoclaved and using sterile tweezers were placed into 24 well culturing dishes (Sigma-Corning). MCF-7 and L929 cells were added to the wells (50,000 cells/well) and cultured as described above in the presence or absence of nanowires. After 24 hr, the cells were moved onto ice were they were washed 3 times with PHEM 1X (10 mM EGTA, 25 mM HERPS, 2 mM MgCl2, 60 mM PIPES and adjusted using NaOH to 6.9) before fixing over night at 4°C with 3.7% PFA. Samples are then rinsed with 1X PHEM 3 times before permeabilising with 0.1% Triton-X in PHEM for 15 min. Samples are then blocked with 5% Goat serum (Sigma) for 30 min and then rinsed 3 times with PHEM before staining with pFAK 397(Santa Cruz Biotechnology). Secondary dyes for Actin and the nucleus were then added for 2 hr before the coverslips were mounted onto a microscope slide and securing with vinyl (Sigma). Con-focal fluorescent imaging was performed using a Zeiss LSM 710 and processed using ZEN lite software.

### SEM analysis of cells grown on WDWs

The silicon wafers were coated by drop casting a thin film of WDWs onto the surface. Cells were cultured on the wafers under standard culturing conditions for 24 hr. The wafers where than rinsed with PBS and placed in 4% PFA for 30 min at 37°C. They were than rinsed with PBS and immersed in 0.5% Osmium tetroxide (Sigma- ReagentPlus) in PBS for 1 hr at room temperature. A dehydration step followed by submersing the sample in (20%, 50%, 75%, 90%, 100%) of ethanol and PBS for 20 minutes at room temperature. Next, samples where immersed in HDMS (Sigma- Corning) for 3 min before gently blotting and storing in a desiccator. Samples were coated with a thin film of gold using a sputter Emitech K550 at 20 mA for 10 sec before viewing on the SEM Hitachi SU-70 at 3 KV.

### Real-time Cell monitoring

Continuous cell monitoring was performed using the xCELLigence system (ACEA) which facilitates label free real-time cell analysis by measuring impedance-based signals across a series of interdigitated gold electrodes. Using E-plates, 100 µL of complete media DMEM was added to each well and the electrodes were allowed to stabilise for 30 min. The plates were then moved into the xCELLigence DP analyzer to set a base line without cells or treatments. MCF-7 cells and L929 cells were then added at relevant seeding numbers (see figure legends) to the plate with varying water dispersible germanium nanowires concentrations. Cells on the electrodes were monitored by reading and recording the cell impedance every 30 min through 100 sweeps.

### LDH assay

MCF-7 and L929 cells were cultured on 96 well plates at 27,000 and 10,000 cells per well respectively. Cells were allowed to adhere to the plate over 8 hr before the media was changed to serum free media to reduce test interference. The membrane integrity was evaluated in the presence or absence of water dispersible nanowires. We used LDH test kits (TOX7 Sigma-Aldrich) and measured membrane integrity by detecting LDH release into serum free media at 1 hr and 24 hr +/− exposure of the cells to nanowires.

### Statistical analysis

Data presented is expressed as mean ± SD. The statistical significance between groups (concentrations and controls) was evaluated using welch t-test and for multiple groups one-way Anova was performed with post hoc test Newmans-Keuls multiple comparison test. All assumptions were tested before performing the statistical tests to insure test significance and compliance. Statistical software R^©^ version 2.15.2.

## Results and Discussion

### Generation and characterisation of water dispersible Germanium nanowires (WDWs)

**Figure 1:**
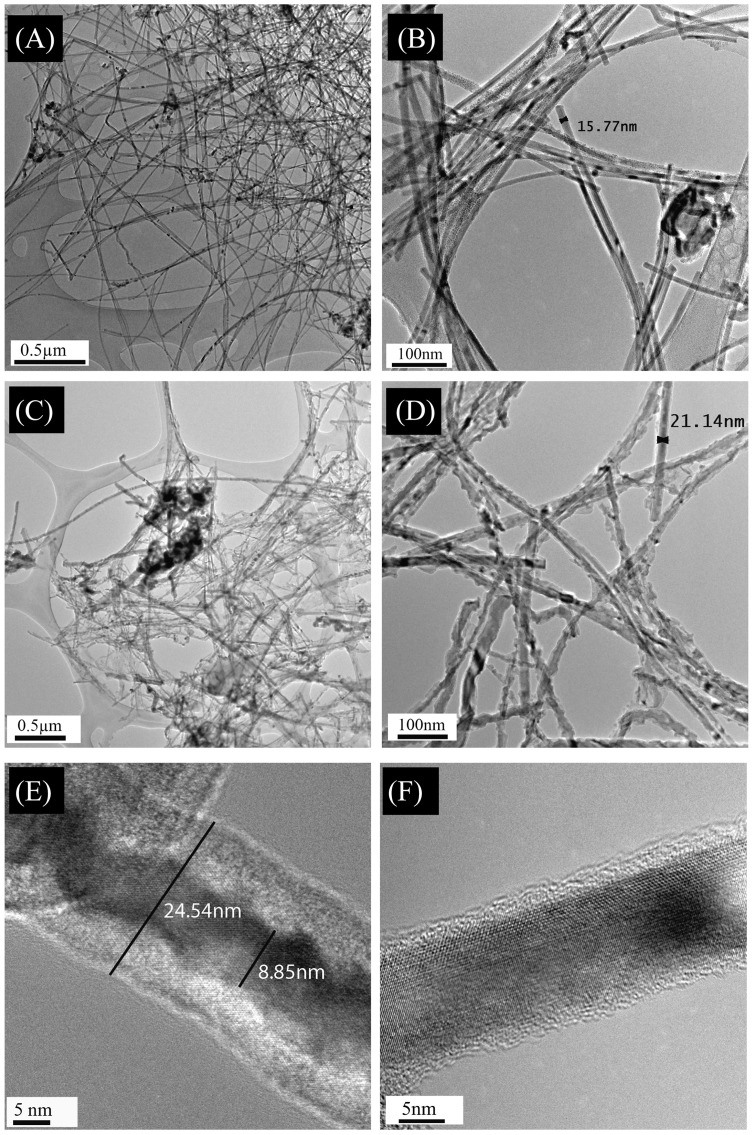
TEM image comparing the surface of pristine unseeded Germanium nanowires and water dispersible Germanium nanowires (WDW) taken on an Electron Microscope JEOL JEM 2100F. (A, B) the high aspect ratio of the pristine Germanium nanowires (GeNW) which span for micrometres. (C, D) the complex surface morphology of water dispersible Germanium nanowires (WDW) after treatment with Glutamic acid, the wires still maintain their aspect ratio span for micrometres. (E) The HRTEM of WDW after 3 hours at 21°C in treatment solution, in comparison to the HRTEM of the pristine wires (F).

Our objective here was to functionalise the wire surface using cell friendly amino acids to facilitate both the dispersion of the wires in aqueous solutions and to increase their biocompatibility over other toxic ligand materials. Figure 1A and 1B show Transmission electron microscopy (TEM) images of pristine wires illustrating the wires are associated with a high aspect ratio. Pristine wires were then sterilized under UV before adding D-Glutamic acid as described in the Materials and Methods. Similar to pristine wires, the treated wires demonstrate a comparable aspect ratio with long undamaged wires extending the length and breadth of the TEM image (Figure 1C and 1D). The images confirm the conservation of high aspect ratio after treatment, suggesting that although the treatment changes the wires surface, it does not degrade the material nor etch it to the point of extensive fragmentation. When the surface of the wires is examined more closely, it is apparent that the treated wires display a distinct surface characteristic which occurs within the first hour of exposure to the amino acid that does not significantly change within the first 24 hr after the treatment (Figure 1E). High resolution TEM (HRTEM) image reveals that after treatment, the wires develop a saw tooth etch surface roughness visible through the increased amorphous coating on the surface (Figure 1E). This surface roughness is dramatic when contrasted against the pristine wire surface which displays a smooth crystalline surface and a notably thinner amorphous coating.

Our observations with the pristine wires showed an inherent tendency for the pristine nanowires to aggregate, however, after treatment this behaviour is significantly reduced, allowing for relatively large areas (cm^2^) to be covered (see scanning electron microscope (SEM) drop casts, Figure S1). These behaviour changes suggest that it is possible to generate a distinct nanomaterial, and not an aggregated nanomaterial, which may prove important as a potential applications of the treated wires.

Using X-ray photoelectron spectroscopy (XPS), we next analysed the surface composition of both pristine wires and the Water Dispersible Nanowires (WDW's). The Ge 2p orbital (Figure S2 A, C) has a higher binding energy than the Ge 3 d orbital (Figure S2 B, D) and was determined to be more representative of the amorphous surface given the lower associated kinetic energy, the Ge 3 d orbital was thus more indicative of the crystalline surface of the material given its higher associated kinetic energy. When WDWs samples were analysed, XPS data revealed that the ratio of germanium oxide to crystalline germanium on WDWs increased after treatment with the amino acid (Figure S2 C, D). However with increased washes with distilled water to remove the amino acid, the oxide to crystalline ratio increased (data not shown). This result highlights the importance of the amino acids to the stability of the surface chemistry of the wire as they prevent the accelerated oxidation of the wires surface in an aqueous environment. The exact affiliation of the amino acid and the wires surface is not clear as any attempts we made to reduce the efficiency of the functional groups on the amino acid by adjusting the pH to the pKa values saw a rapid increase in oxidation ratio (data not shown).

### Water dispersible Germanium nanowires promote cell proliferation and are non-toxic to MCF-7 and L929 cells

**Figure 2:**
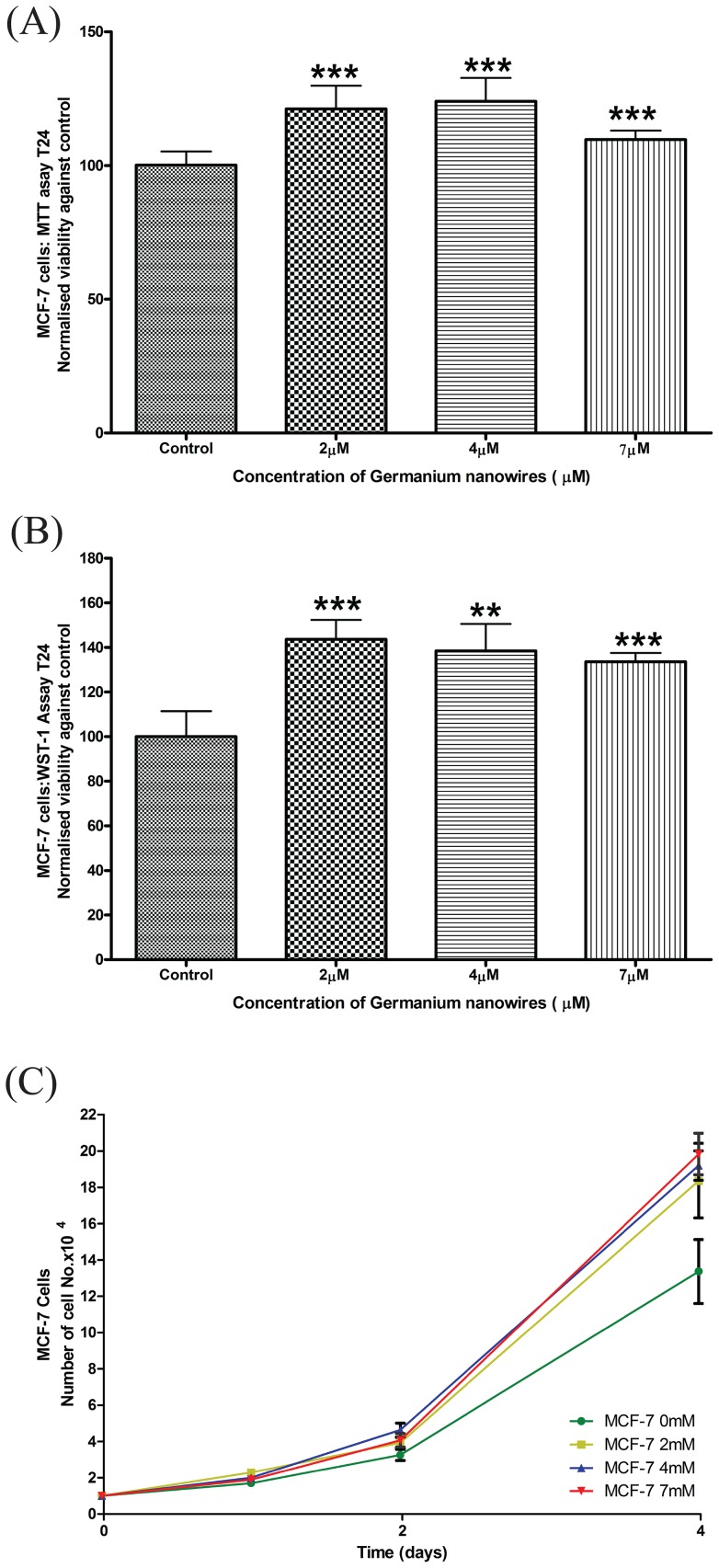
Water dispersible Germanium nanowires promote the proliferation of MCF-7 cells. (A) MCF-7 cells were seeded at 27,000 cells/well, before MTT were carried out over 24 hr as described in the Materials and Methods. Data analysis to determine the level of significance was performed using Welch's t test to determine the level of significance between treatments and control, results were considered to be significant with P<0.05. The results for the above indicate a high degree of significance ***P≤0.001. (B) The normalised viability of MCF-7 cells seeded at 10,000 per well (N>3), was measured after 24 hours of exposure to wires at varying concentrations and measured by WST-1 test. Data analysis was performed using Welch's t test to determine the level of significance.(C) MCF-7 cells were seeded at 10,000 cells/well on increasing concentrations of WDW's for 4 days. Organic cell growth was determined by trypan blue exclusion (n = 5 for each time point and n = 3 for each experiment).

**Figure 3:**
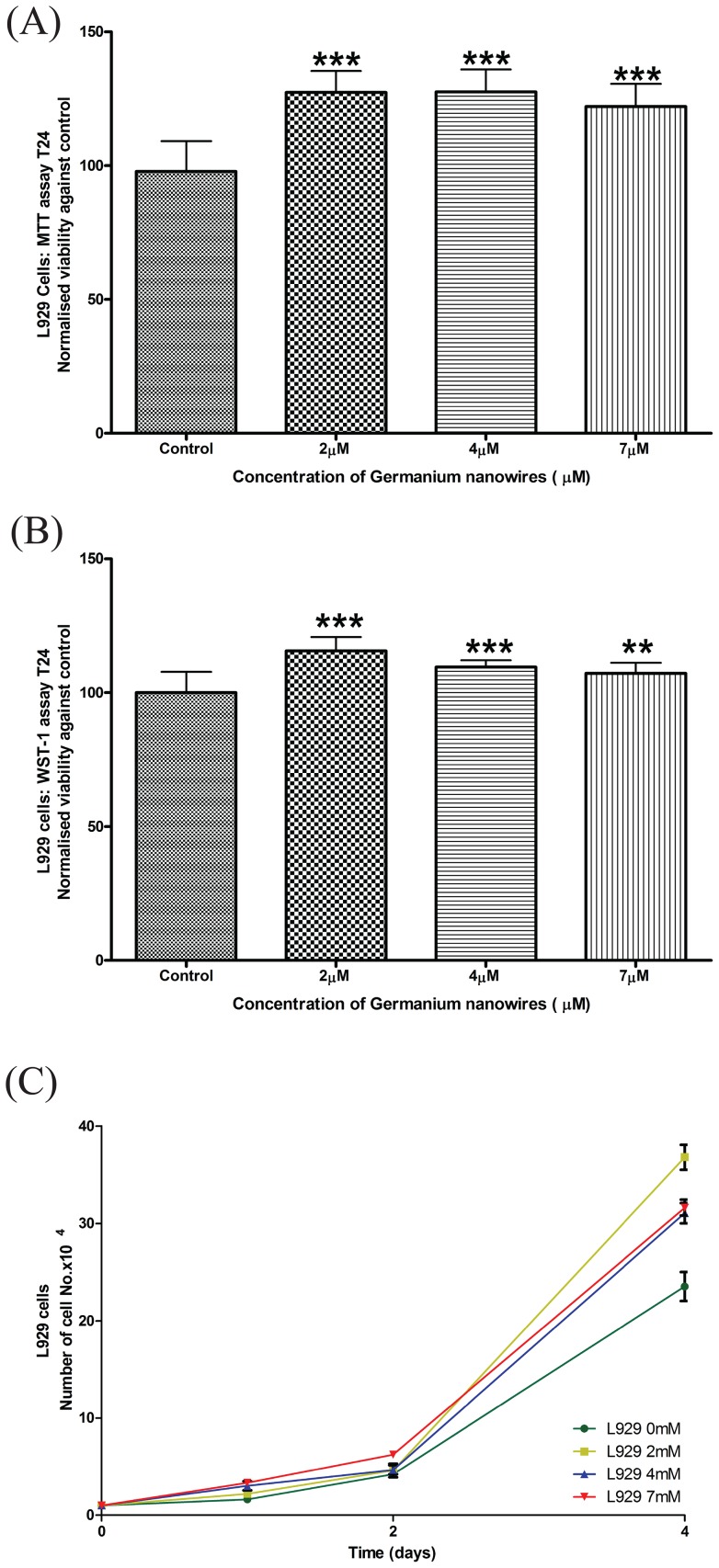
Water dispersible Germanium nanowires promote the proliferation of L929 cells. (A) L929 cells were seeded at 27,000 cells/well, before MTT were carried out over 24 hr as described in the Materials and Methods. Data analysis to determine the level of significance was performed using Welch's t test to determine the level of significance between treatments and control, results were considered to be significant with P<0.05. The results for the above indicate a high degree of significance ***P≤0.001. (B) The normalised viability of L929 cells seeded at 10,000 per well (N>3), was measured after 24 hours of exposure to wires at varying concentrations and measured by WST-1 test. Data analysis was performed using Welch's t test to determine the level of significance. (C) L929 cells were seeded at 10,000 cells/well on increasing concentrations of WDW's for 4 days. Organic cell growth was determined by trypan blue exclusion (n = 5 for each time point and n = 3 for each experiment).

We next set out to validate the use of WDWs as a novel biomaterial and a suitable surface to culture cells. A comprehensive series of state-of-the-art proliferative, cytotoxic, cell viability and behavioural studies were performed as the cells were exposed to bio- relevant concentrations of the WDWs. Previous work with GeNPs used concentration ranges spanning 0–5 µM which we used as the basis for this work [23]. Two well established cell lines; MCF-7 (human breast carcinoma cells) and L929 (murine fibroblasts) were employed for this study as their behaviour has been well studied in our laboratory [35]–[37]. MTT assays were performed using 5 replicates for each concentration of nanomaterial as described in Materials and Methods. To insure that several sets of independent results were obtained, experiments were repeated independently (n = 5) using several different stocks of raw chemical precursors, different nanowire preparations and different cell passages. MCF-7 and L929 cells were grown in the presence of increasing concentrations (2 µM, 4 µM, and 7 µM) of WDW's. The MTT results revealed an increase in the cell viability over control for both MCF-7 cells (Figure 2A) and L929 cells (Figure 3A). The MCF-7 and L929 cells cultured in the presence of WDWs displayed a significant increase in cell viability over respective controls (both with P<0.0001). This increase in viability shows promise towards the use of WDW's as a biocompatible nanomaterial for regenerative applications. The increase in viability may be attributed to the WDWs surface chemistry and topography having an oxide surface with no associated ligands (Figure S2), together with a roughed surface (Figure 1E).

Although the MTT is considered to be the state-of-the-art standard in viability assays, to test the findings further, a series of other well established techniques were employed. The WST-1 test is a colorimetric measurement of cell viability and proliferation. The WST-1 tests were performed in a similar manner as the MTT for both MCF-7 and L929 cells by culturing the cells with increasing concentrations of WDW's for 24 hr before endpoint analysis. The results indicate that when normalised against the controls (showing mean ± SD) that both MCF-7 cells and L929 cells display a significant increase in cell viability against the control when treated with WDWs (Figure 2B, 3B). Data was statistically analysed using a two tailed welch t-test as described in Materials and Methods. In addition to the MTT and WST-1 assays, we performed cell counts on MCF-7 (Figure 2C) and L929 cells (Figure 3C) cultured with increasing concentrations of WDWs (0, 2 µM, 4 µM, 7 µM) for 24 hr before endpoint analysis. To do this, cells were trypsinised from the plate and cell number and cell viability were assessed by the traditional trypan blue exclusion method revealing increased proliferation upon exposure to the WDW's. As well as this, we employed immunofluorescence to examine MCF-7 and L929 cells cultured with increasing concentrations of WDWs (0, 2 µM, 4 µM, 7 µM) for 24 hr before endpoint analysis and staining the nucleus with Hoechst stain (Figure S3, Data shown for MCF-7 cells only). Images clearly show an increase in the number of cells present when treated with increasing concentrations of WDWs. Collectively, these results indicate that WDW's are non-toxic and promote cell proliferation.

### Water dispersible Germanium nanowires promote cell adhesion and increased FAK expression

**Figure 4:**
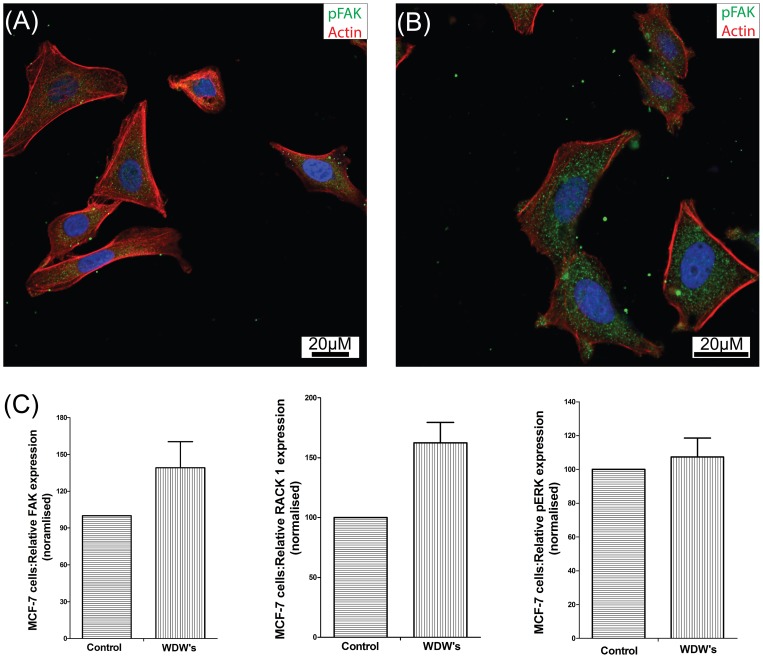
Confocal microscopy to study the morphological features of MCF-7 cells exposed to WDWs. (A) MCF-7 control cells cultured on a 10 µg collagen glass cover slip and stained with phosphorylated FAK (pFAK^397^), Hoechst and Phalloidin TRICI under a 63X oil immersion lens using a Zeiss LSM 710. (B): MCF-7 cells cultured on a 10 µg collagen glass cover slip exposed to 4 µM of Germanium nanowires for 24 hours and stained with phosphorylated FAK (pFAK^397^), Hoechst and Phalloidin TRICI under a 63X oil immersion lens using a Zeiss LSM 710. (C) Dispersible Germanium Nanowires were added to cultures of MCF-7 cells for 24Hr. Lysates were prepared and the lysates ran on 12% SDS PAGE gels before probing with antibodies against Actin (Santa Cruz Biological) pERK (Cell signalling technology phosphor-p44/42 (ERK1/2)), RACK1 (BD Transduction Laboratories) and FAK (Santa Cruz Biotechnology FAK(C-20):sc558). Results were presented as a Histogram of relative FAK expression, RACK1 expression and pERK expression.

**Figure 5:**
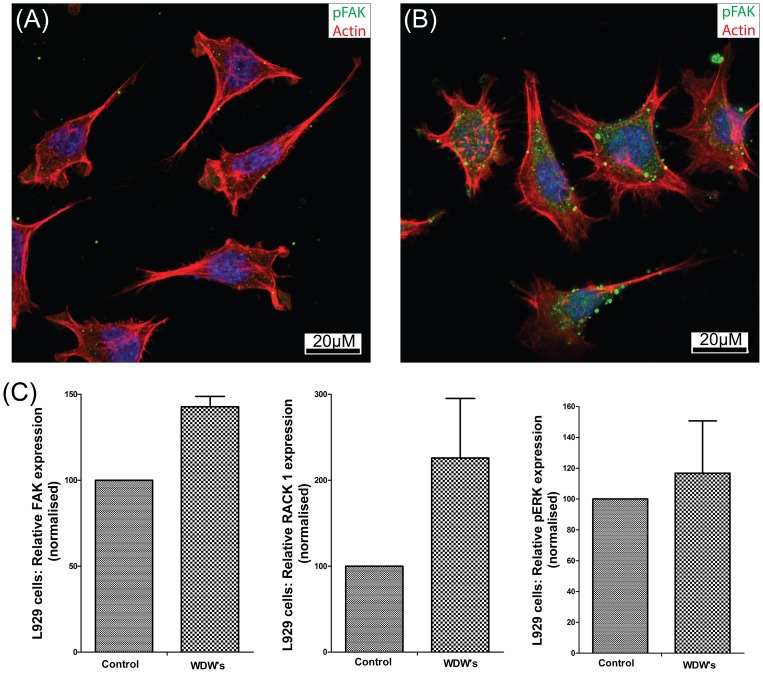
Confocal microscopy to study the morphological features of the L929 cells exposed to WDWs. (A) L929 cells cultured on a 10 µg collagen glass cover slip and stained with phosphorylated FAK (pFAK^397^), Hoechst and Phalloidin TRICI under a 63X oil immersion lens using a Zeiss LSM 710. (B): L929 cells cultured on a 10 µg collagen glass cover slip exposed to 4µM of Germanium nanowires for 24 hours and stained with phosphorylated FAK (pFAK^397^), Hoechst and Phalloidin TRICI under a 63X oil immersion lens using a Zeiss LSM 710. (C) Dispersible Germanium Nanowires were added to cultures of MCF-7 cells for 24 Hr. Lysates were prepared and the lysates ran on 12% SDS PAGE gels before probing with antibodies against Actin (Santa Cruz Biological) pERK (Cell signalling technology phosphor-p44/42 (ERK1/2)), RACK1 (BD Transduction Laboratories) and FAK (Santa Cruz Biotechnology FAK(C-20):sc558). Results were presented as a Histogram of relative FAK expression, RACK1 expression and pERK expression.

Cellular morphology and cell membrane integrity are an important indicator in cytotoxicity and a good indicator of cell health. Studies have shown a decrease in cell adhesion and adverse changes in cell morphology when cells are cultured with silicon nanowires [6], [7]. We performed a series of studies using confocal microscopy to study the morphological features of the cells exposed to WDWs for 24 hr in comparison to respective controls. The MCF-7 and L929 cells were cultured on a glass cover slip coated with 10 µg of collagen I in the presence or absence of WDWs. After 24 hr's, cells were fixed and prepped for staining as described in Materials and Methods. As expected, both MCF-7 cells (Figure 4A control, B WDW's) and L929 cells (Figure 5A Control, B WDW's) display a spread and migratory morphology. We stained with phosphorylated Focal Adhesion Kinase (pFAK^397^) to show that cells grown on collagen/WDWs substrates have increased numbers of focal adhesion. To investigate the changes inside the cell as a result of culturing cells on WDWs, we examined the expression levels of FAK, and phosphorypated ERK1/2, two proteins associated with Integrin and growth factor mediated signalling pathways [35]–[37], and indicators of increased proliferation. The MCF-7 and L929 cells were grown on a 10 cm plate in the presence of wires at different concentrations (see Materials and Methods). The cells were lysed and the protein was separated on a 10% SDS-PAGE gel and transferred to nitrocellulose membrane before probing for FAK and pERK (p42/44). Actin was used as an endogenous control to confirm equivalent amounts of protein were run on the gel. Protein levels of both FAK and RACK1 were expressed at higher levels when the cells were cultured on WDWs (Figure 4C and 5C). High expression of these proteins is associated with increased adhesion and increased proliferation. We also observed consistently increased activation of pERK in cells grown on WDW's. The protein ERK1/2 is a member of the MAPKs family which is involved with several biological process. Increased phosphorylation of Erk is a well-established indicator of cell proliferation. This increased expression and activity of proteins associated with cell adhesion, cell spreading and cell proliferation is further confirmation of the biocompatible properties of the WDW's and provides further confirmation of the MTT and WST-1 assays.

We observed that cells cultured on WDWs show an increased tendency to be directional in growth in comparison to control cells which suggests that the WDW's have potential as to be used as a biomaterial to control directional cell migration. Results shown are representative of hundreds of cells examined (and n>5). We next performed SEM analysis on MCF-7 cells on silicon wafers coated in WDWs (Figure S4). The images suggest that the cells make direct contact with the wires in their surroundings and anchor to the wires. WDWs can be seen all along the base of the MCF-7 cells further illustrating the biocompatibility of the WDWs for the cellular environment. These morphological studies highlight that WDWs are not adversely affecting the cultured cells. It is important to note that we did not detect any membrane blebbing or nuclear ‘break up’ that are associated with cells exposed to toxins and undergoing apoptosis. Long term exposure of the cells to WDW's (up to 72 hr) revealed no further adverse effects on the cells (data not shown).

**Figure 6:**
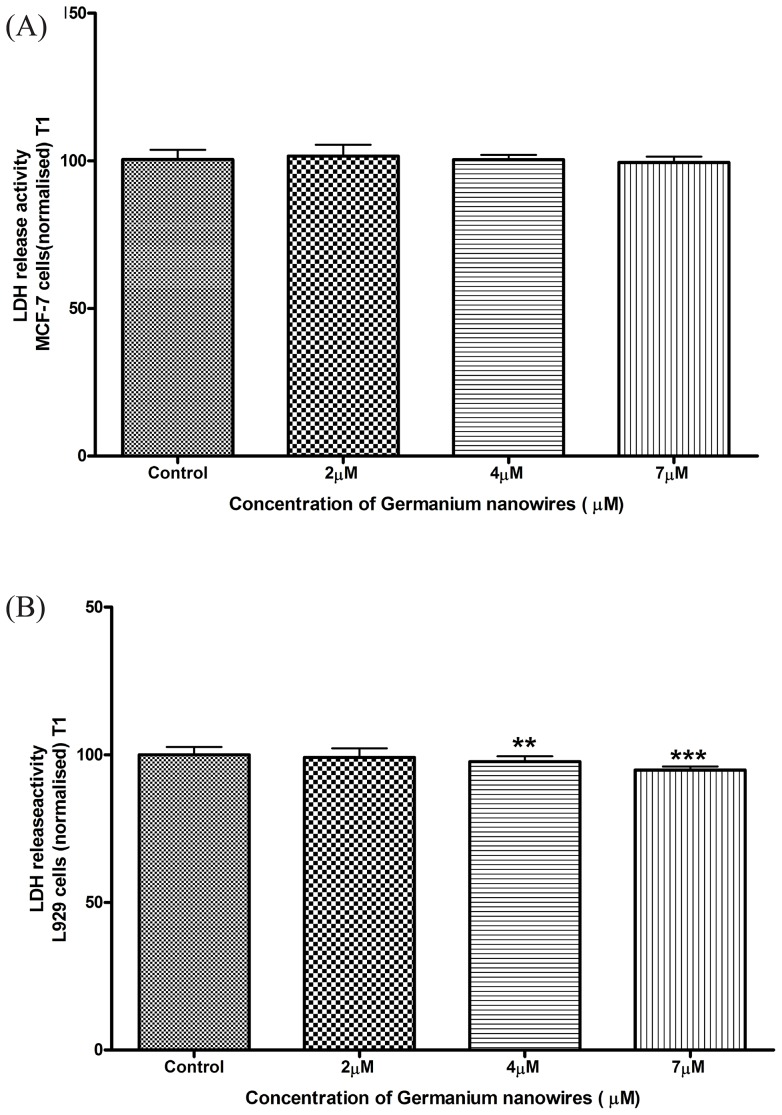
Measuring LDH release into the serum free media when cells are exposed to wires for 1hr. (A): The MCF-7 results at varying concentrations after 1hr, seeded at 27,000 cells/ well, N>3. (B): The L929 results at varying concentrations after 1hr, seeded at 10,000 cells/ well, N>3. Data analysis was performed using one way Anova and Student-Newman-Keuls post hoc test to determine the level of significance between treatments and control, results were considered to be significant with P<0.05. The results for the L929 cells above (B) indicate a significant decrease in the LDH release into the media in which 4 µM displays a mean of 97.68±0.5237 N = 12 **P≤0.01 and 7 µM displays a mean of 94.83±0.3497 N = 12 *P≤0.5.

The data presented in (Figure 4,5) strongly correlates with the MTT and WST-1 results and suggests that the WDWs are not toxic to the cells and provide a biocompatible surface material. In fact, we believe that the WDWs may provide a more suitable surface for spreading than collagen alone. However, with high aspect ratio material there is always a concern that the material will cause membrane damage. To test this, lactate dehydrogenase (LDH) release assays was carried out to evaluate the membranes integrity. The presence of LDH in the growth media is indicative of cell stress caused by cell membrane damage. We recorded the LDH activity present in the growth media after exposure of the cells to the WDW's. The data for both MCF-7 (Figure 6A) and L929 (Figure 6B) are presented as a normalised LDH release against the respective controls. The data plotted clearly shows that for both the MCF-7 cells and L929 cells, LDH release does not increase above the control suggesting that the membrane integrity of the cells is maintained throughout the treatment with WDWs (n = 3). We used two tailed welch t-test, a one-way Anova with post hoc test Newmans-Keuls multiple comparison test which confirmed that these results and findings were statistically significant. Interestingly, for concentrations from 4 µM to 7 µM in L929 cells (Figure 5B) we consistently observed a decrease in the amount of LDH released into the media. This indicates that there is less membrane damage than normal occurring under these conditions suggesting that the WDWs may have a protective effect.

### Real time monitoring of cell behaviour on Water dispersible Germanium nanowires

The results to date provide comprehensive evidence that WDWs are a biocompatible material that promotes cell adhesion and proliferation. We next investigated the changes in the behaviour of the cells as they are grown on WDWs in real-time. To execute this, we employed the xCELLigence system for label-free and real-time monitoring of cell behaviour as cells are grown in the presence or absence of WDW's. This system measures cell adhesion, cell spreading and cell proliferation in real-time by recording cell activity on gold electrodes and correlating the impedance changes as cell index measurements (see materials and methods and [38]–[40]).

**Figure 7:**
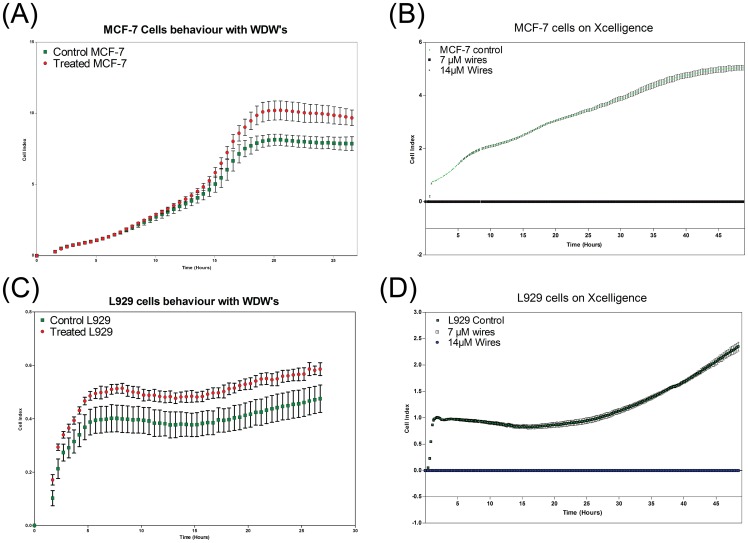
Using live cell impedance based platforms to monitor cell behaviour. xCELLigence (ACEA) platforms were used to monitor cells cultured under the same conditions as those performed in the MTT, WST and LDH assays. All experiments were carried out using E-plates following the ACEA manual protocol where N = 3. (A) Shows MCF-7 cells seeded at 27,000 cells, with and without the presence of WDW (with appropriate controls and blanks). The data indicates that there is an increase in the cell index number a measure of increased impedance across the surface of the plate. (C) Shows L929 seeded at 10,000 cells with and without the presence of WDW (with appropriate controls and blanks). The data indicates that there is an increase in the cell index number a measure of increased impedance across the surface of the plate. (B), (D). MCF-7 cells and L929 cells grown on separate E plates together with increasing concentrations of WDW's to show how the Germanium nanowires themselves have no effect on the impedance as measured by the electrodes.

Results for both MCF-7ccells (Figure 7A) and L929 cells (Figure 7C) were plotted as the mean cell index ± SEM over a 27 hr period. In all cases, samples were run in triplicates and included wells with cells +/− WDWs. We included experiments to control for any effects the nanowires alone might have on the electrode (Figure 7B, D). When MCF-7 cells were grown with WDWs, there is a dramatic increase in the rate of cell proliferation across the surface of the electrode and the cells deviate quickly from the control group with two distinct populations clearly evident after 18hours in culture. This data strongly suggests that the WDWs promotes cell proliferation and correlates well with results seen with the MTT and WST-1 tests (Figure 2 and 3). The increase in adhesion is also correlated to the increase in FAK protein expression seen in (Figure 4C) and a visual increases in morphological adhesion that we observed (Figure 4B) compared to the control in (Figure 4A). The L929 cells (Figure 7C) quickly adhere to the electrode and spread more rapidly than the MCF-7 cells (Figure 7A). Similarly to the MCF-7 cells, there in a dramatic increase in cell index when the L929 cells are grown with WDWs (Figure 7C) correlating well with the MTT, WST-1 and protein data. Taken together, the results provide strong evidence that WDWs promote adhesion, spreading and proliferation of MCF-7 and L929 cells. At no stage did we observe adverse effects of the WDW's on the cells response during our live monitoring (n = 3). This is the first data published using live, label-free cell monitoring to record the behaviour of cells on nanowires.

## Conclusion

As the ever-expanding arena of inorganic nanomaterials primarily focuses on cell tracking and drug delivery, the use of high aspect ratio inorganic nanomaterial has been neglected for bio-regenerative applications. Heretofore, the biocompatibility of nanowires was limited not by the primary material but the associated toxicity of the metal catalysts used in synthesis and/or the organic ligands required during growth or for subsequent dispersion. This work highlights that an appropriate nanowire synthetic strategy that eliminates organic ligands and metal catalysts combined with a benign post synthesis treatment allows for significant benefits in the biological response. Through this body of work we have been the first to show that there are no adverse cytotoxic effects of self-seeded germanium nanowires on mammalian cells. We used a series of synergistic cellular and molecular approaches as well as a series of complimentary cytotoxicity assays to highlight that the WDW's are non-toxic to cells and provide a biocompatible material. To the best of our knowledge, this is also the first paper to show real-time impedance based monitoring of cells on a nanowire surface. These live cell assays indicate that not only have we created a novel biocompatible material, we have produced a material that promotes the adhesion and proliferation of cells.

## Supporting Information

Figure S1 **SEM images are taken on a Hitachi SU-70 at 10KV.** WDWs on a silicon wafer covering a bio relevant area with a simple drop cast with a reduced aggregation.
(TIF)

Figure S2 **XPS results performed on a Kratos AXIS-165.** (A) The 2p binding energy associated with Germanium performed on pristine wires. (B) The 3D binding energy associated with Germanium performed on pristine wires. (C) The 2p binding energy associated with Germanium performed on treated wires. (D) The 3 d binding energy associated with Germanium performed on treated wires.
(JPG)

Figure S3 **Hoechst staining of cells treated with WDWs.** (A) MCF-7 cells control stained after for 24. (B) MCF-7 cells treated with 2 µM WDWs for 24 hours before staining. (C) MCF-7 cells treated with 4 µM WDWs for 24 hours before staining. (D) MCF-7 cells treated with 7 µM WDWs for 24 hours before staining.
(TIF)

Figure S4 **SEM images of an MCF-7 cell cultured on a silicon wafer for 24 hr coated with WDW taken on a Hitachi SU-70 at 3KV.**
(JPG)
