# Supplementary figures and images for: Promoting Cell Proliferation Using Water Dispersible Germanium Nanowires
Source: PLoS One. 2014 Sep 19;9(9):e108006. doi: 10.1371/journal.pone.0108006 (PMC4169628; doi:10.1371/journal.pone.0108006)

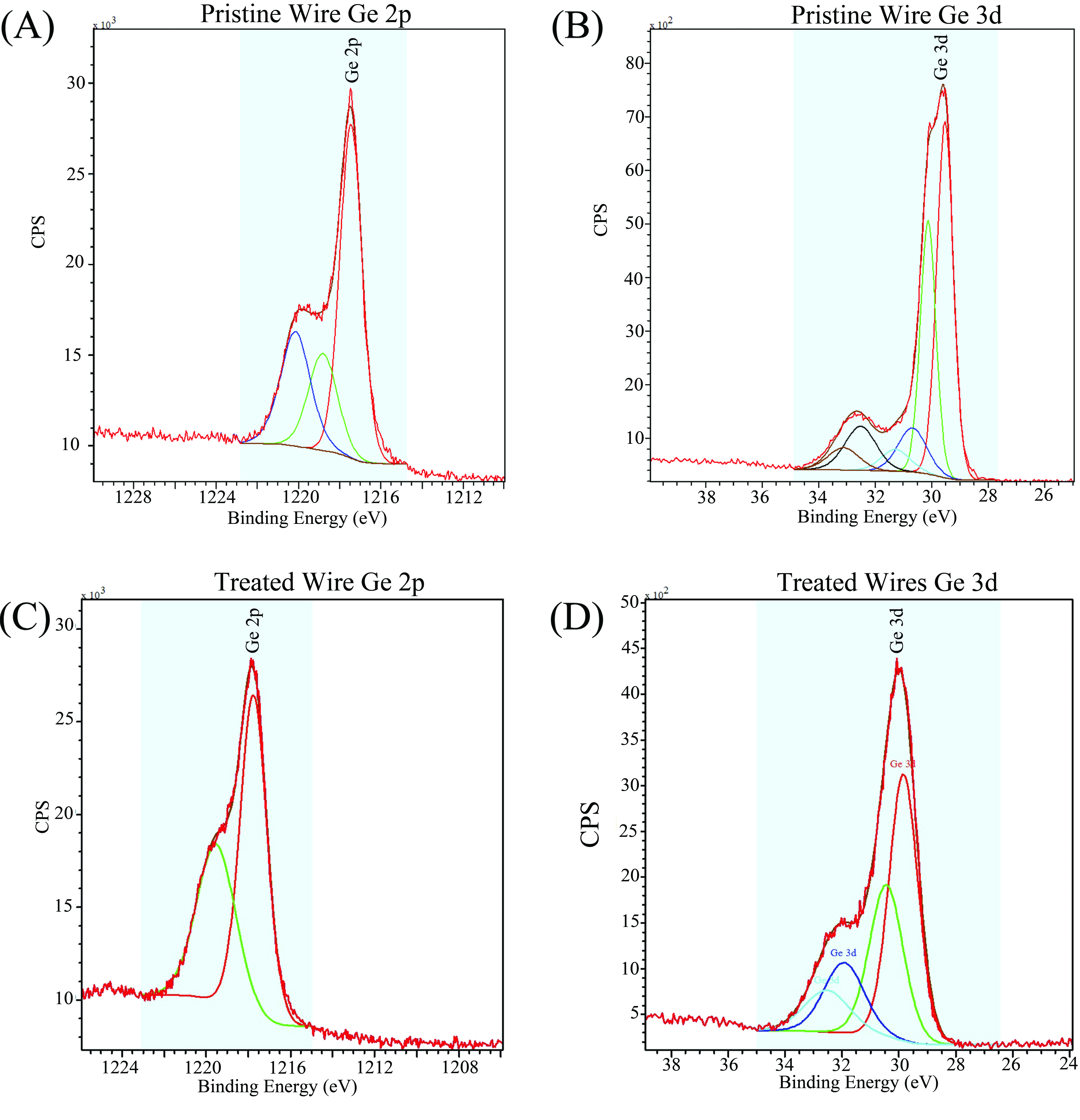

Supplement: Figure S2 — XPS results performed on a Kratos AXIS-165. (A) The 2p binding energy associated with Germanium performed on pristine wires. (B) The 3D binding energy associated with Germanium performed on pristine wires. (C) The 2p binding energy associated with Germanium performed on treated wires. (D) The 3 d binding energy associated with Germanium performed on treated wires. (JPG) [file pone.0108006.s002.jpg]

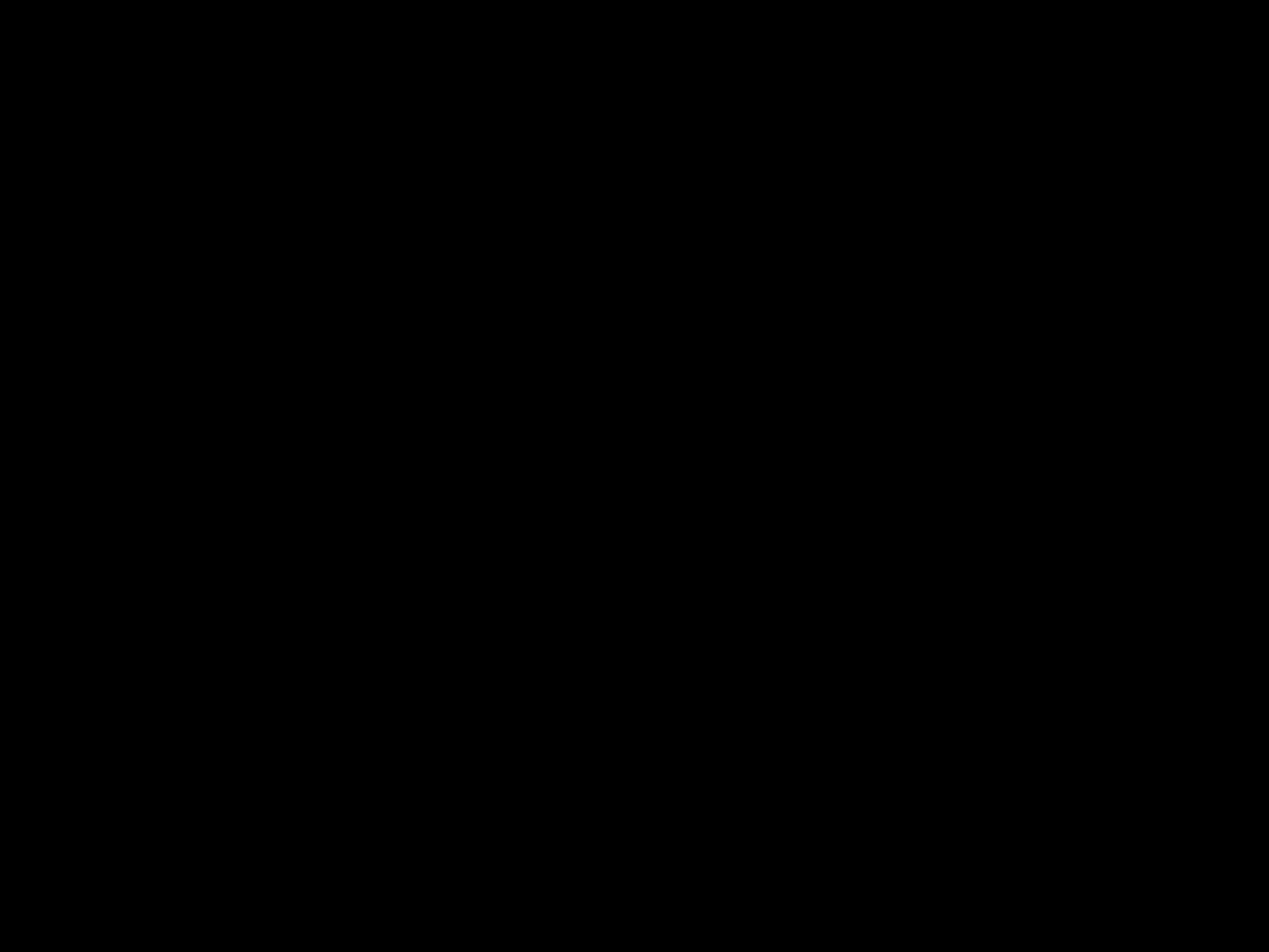

Supplement: Figure S3 — Hoechst staining of cells treated with WDWs. (A) MCF-7 cells control stained after for 24. (B) MCF-7 cells treated with 2 µM WDWs for 24 hours before staining. (C) MCF-7 cells treated with 4 µM WDWs for 24 hours before staining. (D) MCF-7 cells treated with 7 µM WDWs for 24 hours before staining. (TIF) [file pone.0108006.s003.tif]

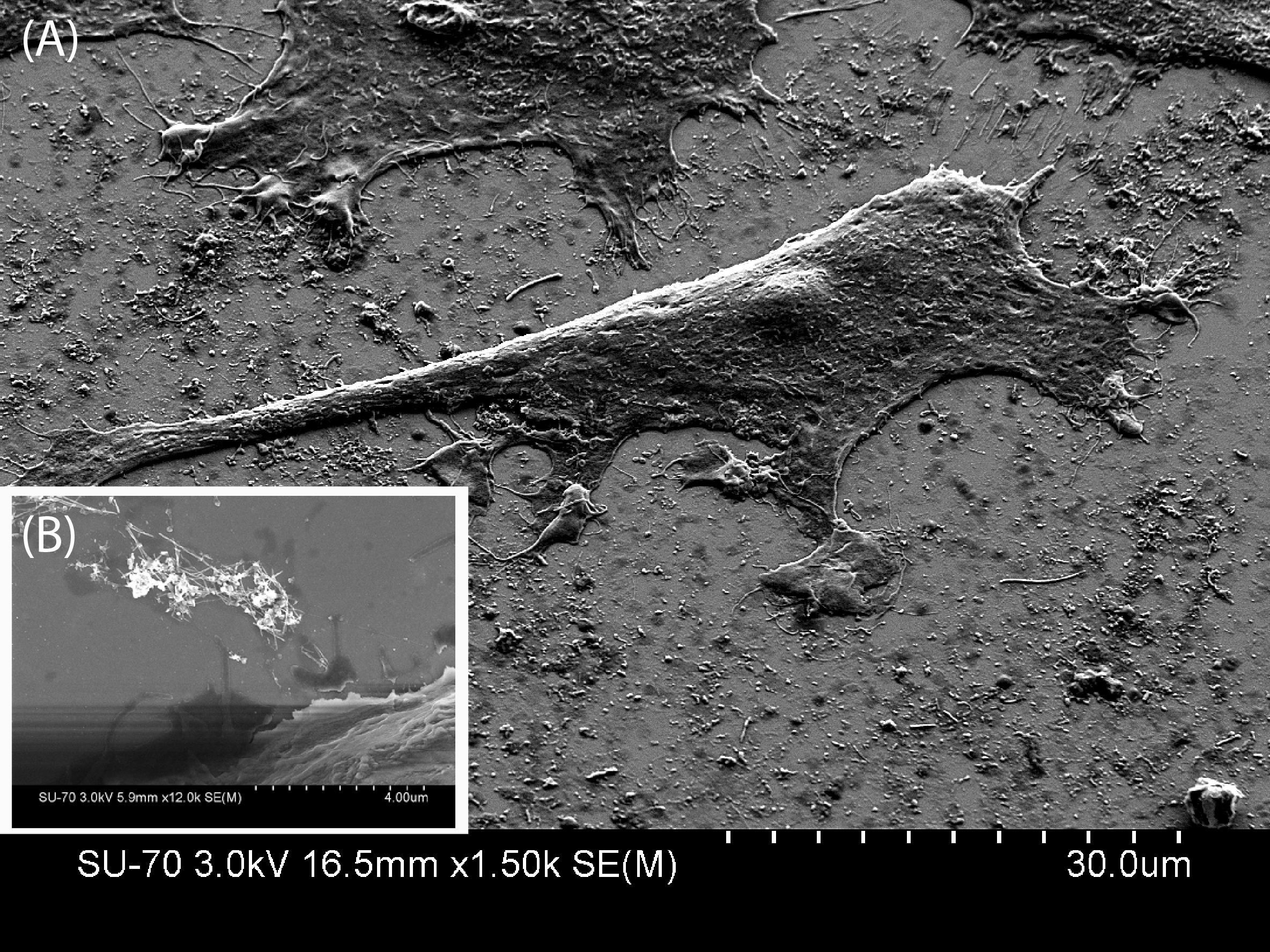

Supplement: Figure S4 — SEM images of an MCF-7 cell cultured on a silicon wafer for 24 hr coated with WDW taken on a Hitachi SU-70 at 3KV. (JPG) [file pone.0108006.s004.jpg]
